# Supplementary material for: Causal interplay between lactose intolerance and gut microbiota: a combined bidirectional Mendelian randomization and in vivo validation study
Source: Front Nutr. 2026 Jun 1;13:1803337. doi: 10.3389/fnut.2026.1803337 (PMC13265576; doi:10.3389/fnut.2026.1803337)
Supplement: Supplementary file 1 [file Data_Sheet_1.zip › supplementary materials/Forward/leave one out cross validation/ebi-a-GCST90027556.finngen_R12_E4_LACTONAS.pdf]

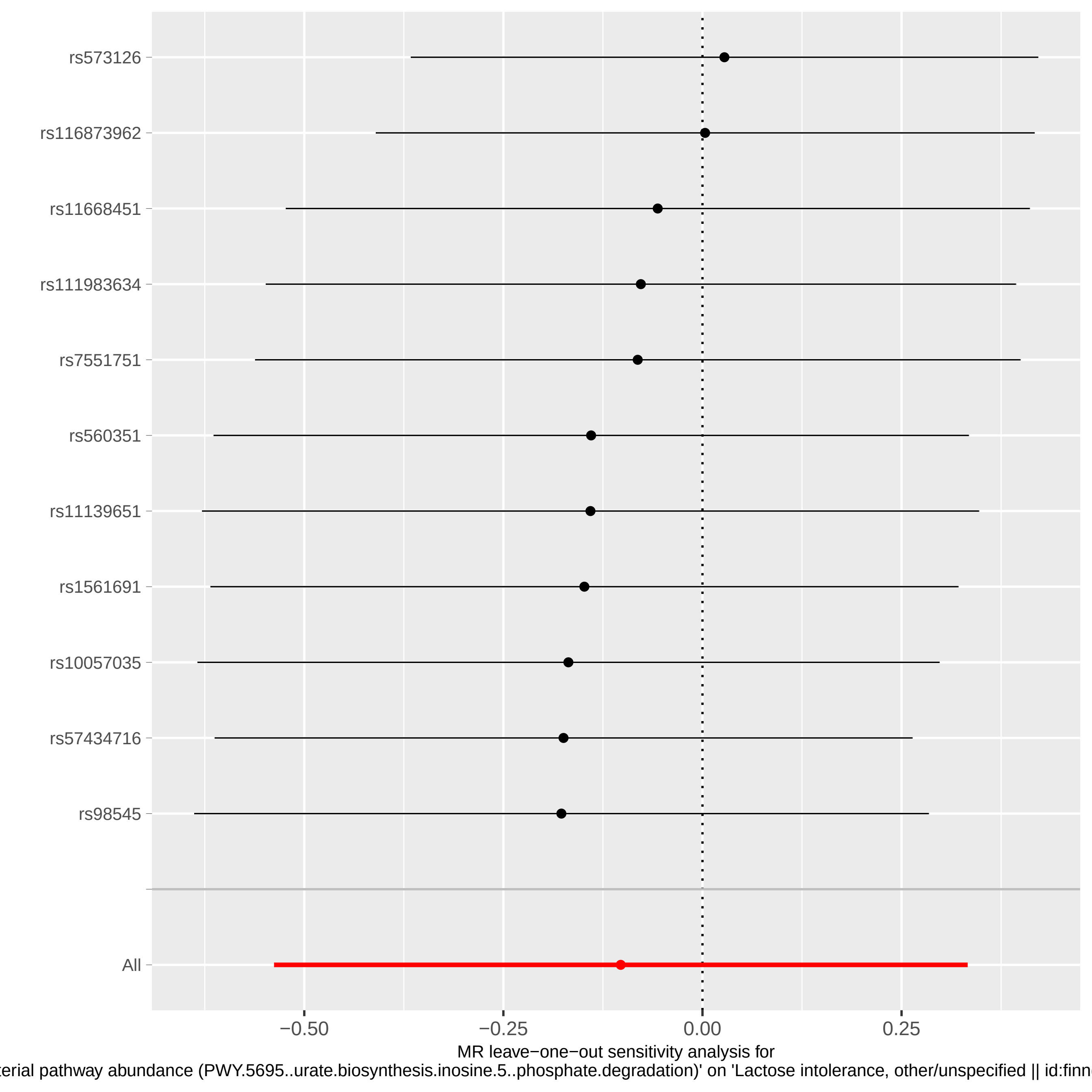
rs573126
rs116873962
rs11668451
rs111983634
rs7551751
rs560351
rs11139651
rs1561691
rs10057035
rs57434716
rs98545
All
−0.50
−0.25
0.00
0.25
MR leave−one−out sensitivity analysis for
terial pathway abundance (PWY.5695..urate.biosynthesis.inosine.5..phosphate.degradation)' on 'Lactose intolerance, other/unspecified || id:finn
